# The impact of the demographic shift on limb amputation incidence in Saskatchewan, Canada, 2006–2019

**DOI:** 10.1371/journal.pone.0274037

**Published:** 2022-09-02

**Authors:** Samuel Kwaku Essien, Audrey Zucker-Levin

**Affiliations:** School of Rehabilitation Science, University of Saskatchewan, Saskatoon, SK, Canada; University of Illinois at Chicago, UNITED STATES

## Abstract

**Background:**

Changing demographics in a population may have an inevitable influence on disease incidence including limb amputation. However, the extent to which these changes affect limb amputation (LA) is unknown. Understanding the impact of changing demographics on LA would provide the best opportunity to plan for the future. We assessed the impact of changes in age and sex on limb amputation in Saskatchewan between 2006 and 2019.

**Methods:**

Retrospective linked Saskatchewan’s LA cases, and demographic characteristics and residents population from 2006–2019 was used. The amputation rate was calculated by dividing the total number of LA cases recorded each year by the annual Saskatchewan resident population and the results expressed per 100,000 populations. Furthermore, decomposition analysis was used to assess the impact of changes in age and sex on LA in a decade (2008–2017) and the Generalized Additive Model (GAM) was employed to examine the linear and non-linear effect of age.

**Results:**

We found that in the ten years (2008–2017), the absolute LA rate difference was 9.0 per 100,000 population. Changes in age structure alone contributed 7.7% to the LA rate increase and 92.3% to changes in age-specific LA rates. The decade witnessed a marginal population difference between males and females, but the LA rate was 2.1–2.2 times higher in males than in females. The GAM revealed a non-linear relationship between LA and age, and further indicates that the risk of LA significantly increased as age increases.

**Conclusions:**

In a decade, we found that changes in age distribution and age-specific rate substantially impacted the increase in the LA rate observed in the province. This highlights the urgent need for strategized programs to respond to these changes as both the population and diabetes, which is age-dependent and a leading cause of LA, are expected to increase in the province by 2030. As changes in population and demographic factors are inevitable, this study provides data for policy makers on the need for continuous incorporation of the shift in population in the design of future health services.

## Introduction

Changes in the size of a given population may be attributed to the change in the magnitude of in-migration, out-migration, births, and deaths [1, 2]. These epidemiologic transitions have an inevitable influence on the distribution of population characteristics such as age [2]. A particular concern is the influence that the changing demographics may have on disease incidence [3–5]. Not only do these changes affect the demographic and disease profiles, but they may alter the health care systems’ capacity and capability to respond to these changes [5].

Despite an overall increase of 5.9% in population observed in Canada between 2006 and 2011 [6], the rate of population growth in the same interval increased by 6.7% in Saskatchewan [6]. These populations’ shifts may have impacted Canadian jurisdictions differently on many age-related impairments, including limb amputation (LA) [7]. A Canadian national study published by Imam et al. identified the age-specific rate of LA for persons aged 75+ years increased by 1.42% between 2006 and 2008 but decreased by 6.47% among 50–74 years [7]. However, between 2008 and 2011, the rate among persons aged 75+ years rather declined by 8.49%, whereas no change in the rate was observed among those aged 50–74 years [7]. Besides, changes in population demographic composition may consequently influence LA incidence and rates among males and females [8]. Cai et al. found that a change in demographic composition from 2008 to 2018 led to an increased proportion of women veterans but resulted in decreased incidence of lower extremity amputation [8].

As 25% of the Canadian population is expected to be 65 years of age or older by 2036 [9], a better understanding of how the most recent population shifts contributed to changes in the incidence of LA would be helpful in preparing for future shifts in demographic factors. This leads to the question: “How much of the absolute difference between the highest and the lowest LA rates in Saskatchewan is attributable to changes in the population’s age and sex distributions?”

Moreover, the implication of these changes on the shape of association (linear vs. non-linear) between age and LA incidence over time is not known. Inferentially, the shape can be tested using linear models [10] but is limited in elucidating covariates’ non-linear effect [11]. Imposing linear models on a covariate with a non-linear effect may undermine both the fit’s goodness and hinder understanding of the complex association between the covariate and the outcome variable [12]. Hence, assessing the shape of the relations may help to understand and elucidate the potential effect of age on LA, which would provide baseline information for future optimal method selection in this research line. Therefore, we assessed (1) the impact of the contribution of age and sex structural transition on the incidence of LA in Saskatchewan using decomposition analysis and (2) the comparative significance of the linear and non-linear effect of age on LA incidence using the Generalized Additive Model (GAM).

## Methods

### Data

Annual provincial retrospective data based on hospital discharge amputation intervention codes (1SN93, 1SQ93, 1TA93, 1TK93, 1TM93, 1TV93, 1VA93, 1VC93, 1VG93, 1VQ93, 1UB93, 1UE93, 1UF93, 1UG93, 1UH93, 1UI93, 1UJ93, 1UK93, 1UM93, 1WA93, 1WE93, 1WI93, 1WJ93, 1WK93, 1WL93, 1WM93, 1WN93) [13] were obtained from eHealth Saskatchewan through the Saskatchewan Health Quality Council. The data included all LA cases recorded in the province from January 1, 2006, to December 31, 2019. Besides, personal factors, including the age and sex of each patient, were retrieved from the *Person Health Registration System*, which was then merged to the data file containing the LA cases. The accuracy and completeness of these administrative databases have popularized their continuous utilization in most population health studies [14–16]. In addition, population data were retrieved from 2006 to 2019 from the Saskatchewan Bureau of Statistics [17]. This study applied and received Ethical approval from the University of Saskatchewan Biomedical Ethics Board (U of S # Bio 1590).

### Data analysis

The amputation rate was calculated by dividing the total number of LA cases recorded each year by the annual Saskatchewan resident’s population and the results expressed per 100,000 populations. Furthermore, a decomposition analysis was performed to assess and quantify the absolute LA rate difference between the highest and the lowest LA rates attributable to changes in age-specific rate and age distribution [1]. The decomposition procedure stipulated and described in Preston (Preston et al., 2001, p.28) [1] was used. The procedure commenced by assigning *ACR^lowest^* and *ACR^highest^* to years with the lowest and highest amputation crude rates, respectively. Also, we let ${ACR}_{i}^{lowest}$ and ${ACR}_{i}^{highest}$ represent age-specific rates of years with lowest and highest LA rates, where *i* denotes age groups considered (*i* = 0–49 years, 50–64 years, 65–74 years, and 75+ years). Additionally, we represented years Saskatchewan’s population’s age distributions corresponding with years with the lowest and highest LA rates as $P_{i}^{lowest}$ and $P_{i}^{highest}$, respectively. All the decomposition analyses were carried out via the following formulas:

Contribution as a result of changes in the age distribution (A)

$$\sum_{i}\left(P_{i}^{highest}-P_{i}^{lowest})*[\frac{{ACR}_{i}^{highest}+{ACR}_{i}^{lowest}}{2}\right]$$

Contribution as a result of changes in age-specific amputation rate (B)

$$\sum_{i}\left({ACR}_{i}^{highest}-{ACR}_{i}^{lowest})*[\frac{P_{i}^{highest}+P_{i}^{lowest}}{2}\right]$$

The total contribution due to both changes in age distribution and age-specific amputation rate (C)

$$\sum_{i}\left(P_{i}^{highest}-P_{i}^{lowest})*[\frac{{ACR}_{i}^{highest}+{ACR}_{i}^{lowest}}{2}\right]+\sum_{i}\left({ACR}_{i}^{highest}-{ACR}_{i}^{lowest})*[\frac{P_{i}^{highest}+P_{i}^{lowest}}{2}\right]$$

Proportions due to changes in age distribution and age-specific rate were estimated from $\frac{A}{C}$*100 and $\frac{B}{C}$*100, respectively.

For the ease of interpretation, the outcome of a given age group with a negative value of age composition, but a positive value of age specific rate is an indication that both decomposed factors worked in the opposite direction of the other [1].

To understand and elucidate the potential effect of age (0–49 years represented by 1, 50–74 represented by 2 and 75+ represented by 3) on the incidence of LA, a Poisson Generalized Additive Model (GAM) [18, 19] was used to linearly model age and year of amputation. The B-spline function was applied [18] to model and identify the potential non-linear effect of age and year of amputation on LA rate [18, 19]. The strength of the no-linear relationship between the LA rate and covariates was quantified using the effective degrees of freedom (EDF) of GAM [20, 21], with EDF = 1 signifying a linear relationship and EDF>1 representing a non-linear relationship [20, 21]. The higher the EDF, the higher the non-linear relationship [21].

In the model building process, a variable with a p-value less than 0.25 in the unadjusted model was included in the adjusted model. A manual backward elimination technique was employed to select significant variables (p<0.05) for the final adjusted model. Also, overdispersion was assessed and compared between Poisson and negative binomial regression. Based on the Akaike Information Criterion (AIC) [22], no evidence of overdispersion was detected.

The AIC was again employed to compare the two models’ optimal performance (Poisson generalized additive model with non-linear effect of age and year of amputation vs. Poisson generalized additive model with linear effect of age and year of amputation), and the model with the smallest AIC deemed to fit the data much better [23]. Moreover, the analysis of deviance was carried out to assess the model fit further. Thus, a model with the smallest residual deviance was considered the best-fitted model.

## Results

**Fig 1:**
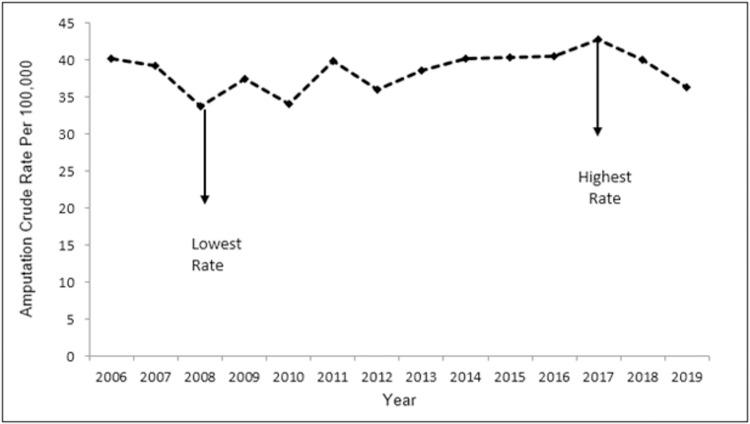
Plot depicting the lowest and the highest overall amputation LA rates in Saskatchewan, 2006–2019.

**Fig 2:**
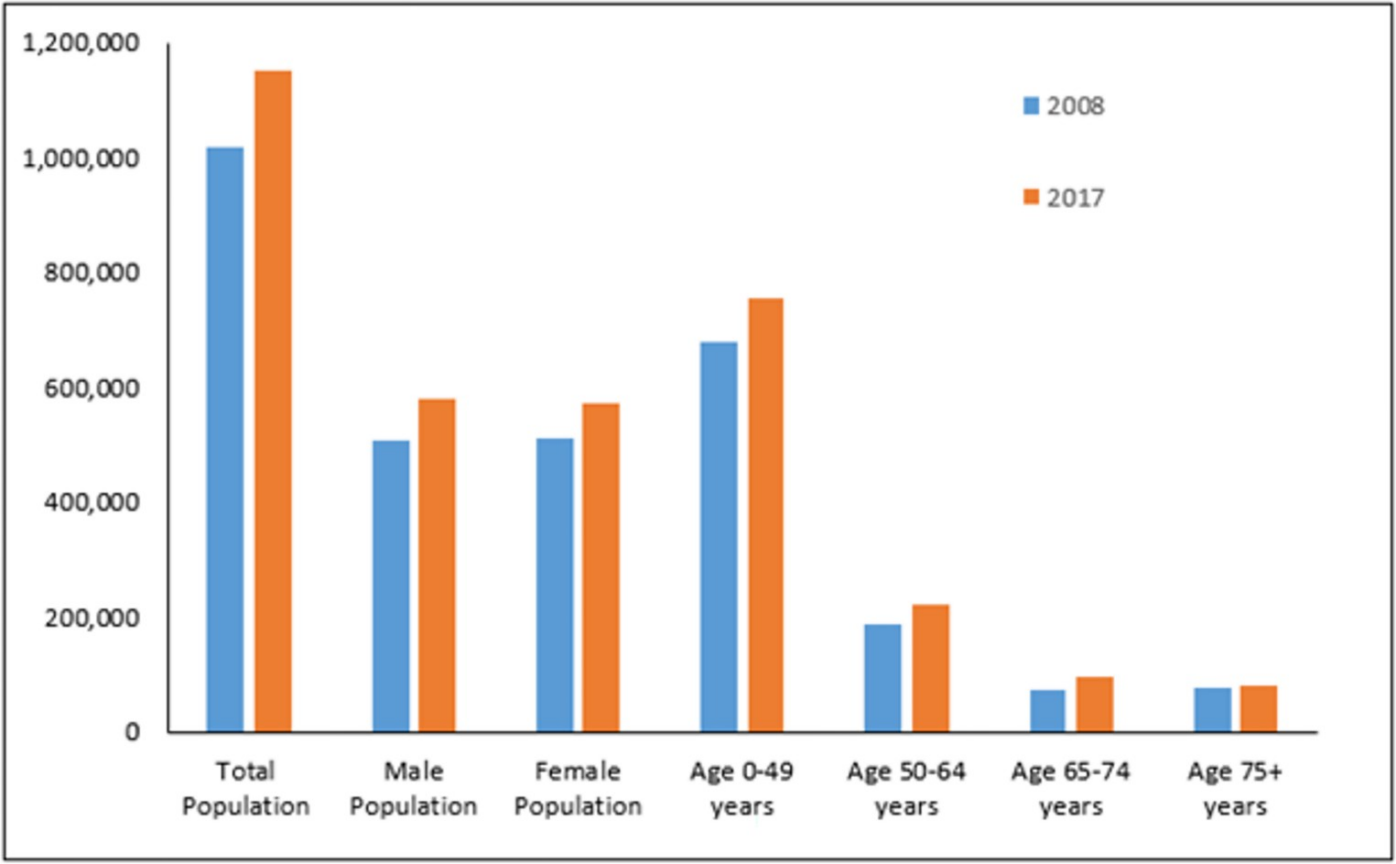
Comparison of population distribution between 2008 and 2017 in Saskatchewan.

Over the past 14 years, the rates of LA have fluctuated, with the lowest and highest rates observed in 2008 (33.8 per 100,000) and 2017 (42.8 per 100,000), respectively. This resulted in an absolute LA rate difference of 9.0 per 100,000 (Fig 1). However, this rate difference is complicated by a concomitant Saskatchewan population increase of approximately 13.1% (rate of 1.31% annually) (Fig 2). During this period, the total population change was not equally distributed when age and sex are examined, impacting the overall change in age group and sex distribution in the population. From 2008–2017 the population of Saskatchewan increased in all examined age groups approximately as follows: 10.8% in persons aged 0–49 years (rate of 1.08% annually); 19.1% in persons aged 50–64 years (rate of 1.91% annually); 31.64% in persons 65–74 years (rate of 3.16% annually); and 0.96% in persons75+ years (rate of 0.01% annually).

In addition, the male population increased approximately 14.5% (rate of 1.45% annually) while the female population increased 11.6% (rate of 1.16% annually) in the same decade interval.

### The male-female case-to-population ratio in 2008 and 2017

Table 1 compares the case-to-population ratio for males and females in 2008 and 2017. Comparing the male-female case-to-population ratio in 2008 revealed that the male LA rate was 2.1 times higher than the female. This was consistent with the 2.2 times higher male-to-female case-to-population ratio in 2017.

**Table 1:** Overall amputation case—to-population ratio.

Sex	2008 Amputation Cases	2008 Population	Case-to-Population Ratio	2017 Amputation Cases	2017 Population	Case-to-Population Ratio
**Female**	110	511422	0.00022	154	570832	0.00027
**Male**	234	505982	0.00046	338	579499	0.00058

### The impact of changes in age distribution in the entire cohort and male cohort on LA rates

Table 2 assess the impact of changes in age distribution and age-specific LA rate on the absolute difference in the overall LA rates in 2008 and 2017. The results show that the LA rate difference in 2008 (33.81 per 100,000) and 2017 (42.77 per 100,000) yielded an absolute increase of 9.0 per 100,000. Decomposition by age category alone attributed 7.7% of the increase to changing population age structure and 92.3% to a changing age group-specific LA rates. The age group with the largest contribution of age compositional difference was 65–74 years, while the age group with the largest contribution of age-specific rate difference was 50–64 years.

**Table 2:** The contribution of changes in age group distribution and age-specific rates on LA.

**Entire Study Cohort**										
	**2008**				**2017**				**Contribution**	
**Age/years**	**Cases**	**Population**	**Rate/100,000**	**Age Comp**	**Cases**	**Population**	**Rate/100,000**	**Age Comp**	**Age Comp Difference**	**Age-Specific Rate Difference**
0–49	79	679830	11.62	66.8%	121	753548	16.06	65.5%	-18.17	293.56
50–64	99	187186	52.89	18.4%	161	222916	72.23	19.4%	61.31	365.23
65–74	80	71811	111.40	7.1%	102	94535	107.90	8.2%	127.17	-26.79
75+	86	78577	109.45	7.7%	108	79332	136.14	6.9%	-101.53	195.10
**Total**	**344**	**1017404**	**33.81**	**100%**	**492**	**1150331**	**42.77**	**100%**	68.78	827.10
**Proportion**									**7.7%**	**92.3%**
**Male Cohort**										
	**2008**				**2017**				**Contribution**	
**Age/years**	**Cases**	**Population**	**Rate/100,000**	**Age Comp**	**Cases**	**Population**	**Rate/100,000**	**Age Comp**	**Age Comp Difference**	**Age-Specific Rate Difference**
0–49	62	345945	17.92	68.4%	77	386526	19.92	66.7%	-31.62	135.01
50–64	77	94283	81.67	18.6%	118	113289	104.16	19.5%	85.09	429.36
65–74	55	34337	160.18	6.8%	74	46827	158.03	8.1%	205.94	-15.97
75+	40	31417	127.32	6.2%	69	32857	210.00	5.7%	-90.94	491.09
**Total**	**234**	**505982**	**46.25**	**100%**	**338**	**579499**	**58.33**	**100%**	168.47	1039.49
**Proportion**									**14.0%**	**86.0%**

Age comp = Age compositional; Outcome of Equation A = Total value of Age compositional difference; Outcome of Equation B = Total value of Age-specific rate difference; Outcome of Equation C = Outcome of Equation A + Outcome of Equation B.

In addition, Table 2 also revealed an absolute increase of 12.08 per 100,000 in LA rate between male cohort LA rate in 2008 (46.25 per 100,000) and 2017 (58.33 per 100,000). The decomposition analysis further showed that 14.0% of the increase in the absolute rate difference among the male cohort was attributable to changes in age group distribution during the ten years (2008–2017), and 86.0% was attributable to changes in age-specific LA rate. More so, consistent with the entire cohort’s finding, males aged 65–74 years produced the largest contribution of age compositional difference. In contrast, the male cohort aged 75+ was the largest contribution of age-specific rate difference.

### Assessing the potential effect of age on the incidence of LA

Table 3 compares the Poisson generalized additive model results, which incorporated the non-linear effects of age and year of amputation, to a Poisson generalized additive model with linear effects of age and year of amputation.

**Table 3:** Models assessing the linear and non-linear effect of age.

**Covariates**	**Model I**: Poisson generalized additive model with non-linear effect of age								
	Estimate	Standard Error	P-value	Relative Risk (RR)	95% CI	AIC	Resid.Df	Resid.Dev	Pr(>Chi)
**Sex**						8956.13	89.3	1679.0	<2.2e-16***
**Female (ref)**									
**Male**	0.897	0.0085	<2e-16***	2.45	(2.41–2.49)				
**Smooth Covariate**	Approximate Significance of Smooth Terms								
	Edf	Ref.df							
**Age**	1.994	2.000	<2e-16***						
**Year**	7.909	8.687	<2e-16***						
Year*Age	9.994	9.999	<2e-16***						
	**Model II**: Poisson generalized additive model with linear effect of age								
	Estimate	Standard Error	P-value			AIC	107.0	2851.9	
**Sex**						10095.26			
**Female (ref)**									
**Male**	0.898	0.0090	<2e-16***						
**Age**	27.216	2.7084	<4e-16***						
**Year**	0.046	0.0028	<2e-16***						
Year*Age	-0.014	0.0013	<2e-16***						

Edf-Estimated degrees of freedom; Ref.df-Degrees of freedom before smoothing; Chi.sq-Chi-square value Significance level = ‘***’ 0.001; Resid.Df-Residual degrees of freedom; Resid.Dev-Residual deviance; Pr (>Chi) -Associated p-value corresponding to the Chi-square test; RR-Relative Risk; CI-Confidence Interval.

Irrespective of all three covariates demonstrated a statistically significant association with LA incidence for both models, the Poisson generalized additive model with non-linear effect of age and year of amputation (*Model I*) produced the smaller AIC (8956.13) compared with the AIC (10095.26) from the Poisson generalized additive model with linear effect of age and year of amputation (*Model II*). Since smaller AIC indicates a much better fit of the model, this suggests that incorporating non-linear effects of age and year of amputation improved the model fit much better than the linear fixed effect of age and year of amputation.

More so, the R-square from *Model I* (the proportion of the variance in the LA rate accounted for by the covariates) was 88.0%, and that of *Model II* was 79.5%.

Further statistical evidence from the deviance analysis revealed that *Model I* had a much smaller residual deviance (Resid.Dev = 1679.0) compared with its counterpart *Model II* (Resid.Dev = 2851.9). It is worth noting that the smaller the residual deviance, the better the model is deemed to fit the data well. This suggests that the *Model I* produced the best fit supported by a p-value of 2.2e-16 at a significance level of 0.001. Further, from the *Model I*, age had an effective degrees of freedom EDF = 2.0, which was greater than EDF = 1.0 for a linear effect. This clearly demonstrates that the underlying relationship between age and LA incidence is nonlinear.

Hence, based on the results from the model comparison/selection (see Table 3), all subsequent detailed analyses and interpretations were carried out using *Model I*.

**Fig 3:**
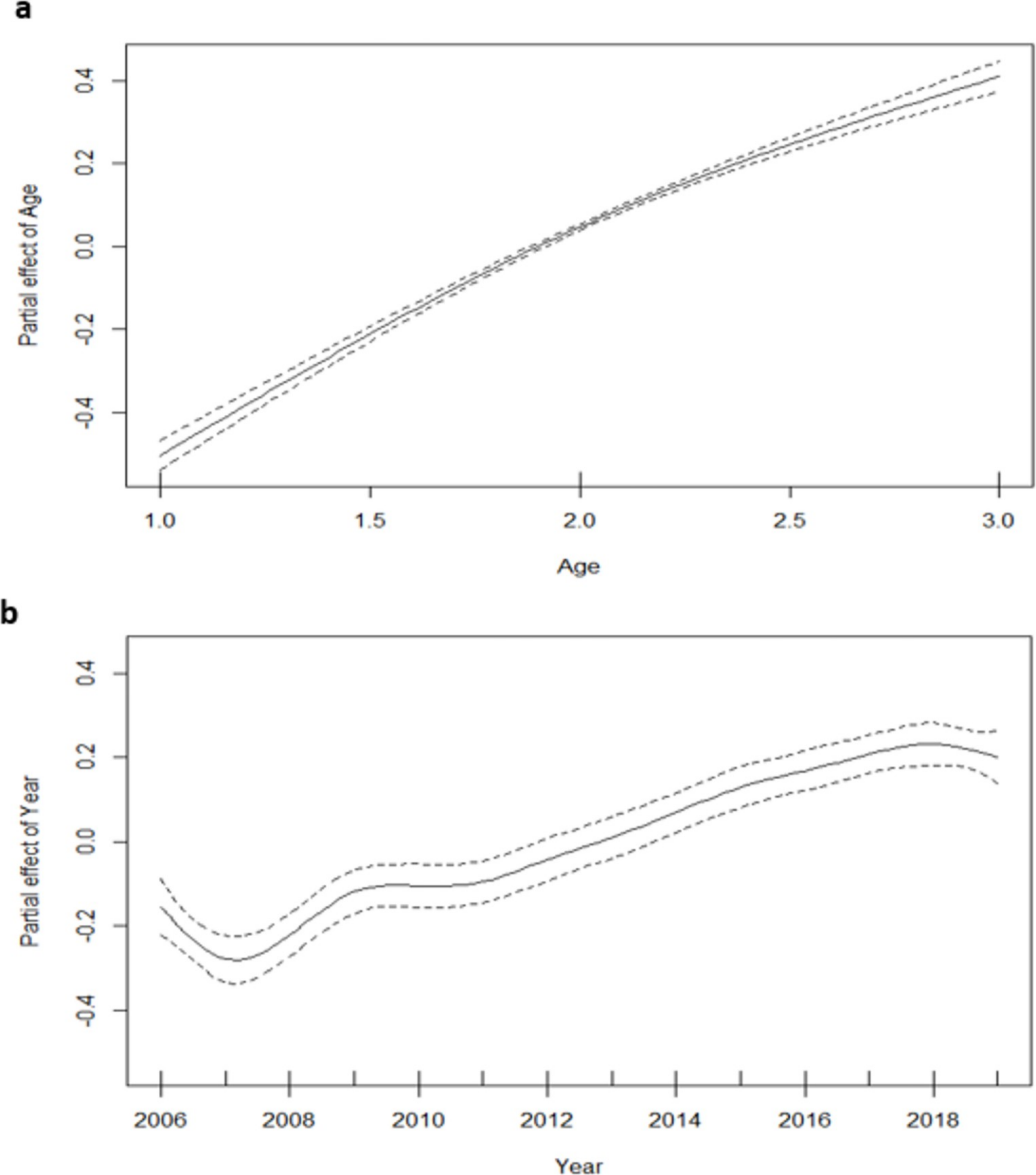
Plot of non-linear effects of age and year of amputation on limb amputation risk. The solid black lines represent the estimated non-linear effects of the log relative risk of limb amputation, and the interrupted lines represent the corresponding 95% Confidence Interval of the estimated risk. Age 0–49 years = 1.0, 50–74 years = 2.0 and 75+ years = 3.0.

According to *Model I* in Table 3, the results show that sex was significantly associated with LA, with a 2.4-fold greater risk of LA in males than females (RR = 2.45, 95% CI: 2.41–2.49). Fig 3 portrays the effects of age and year of amputation on LA rate. Fig 3(A) shows a non-linear relationship between the risk of LA and the age of patients, and it further indicates that the risk of LA significantly (p<2e-16) increased as age increases. Fig 3(B) shows that LA’s risk declined moderately from 2006–2007 and increased consistently from 2007–2018 before finally experiencing a slight drop between 2018 and 2019. In addition, the interaction between age and year of amputation was found to be significant, according to Table 3.

## Discussion

Using the decomposition method to assess the contribution of population and age structural transition on LA, we found that in the ten years (2008–2017), among the entire cohort, the increase in absolute LA rate difference was 9.0 per 100,000 population whereas the male cohort had an absolute LA rate difference of 12.0 per 100,000 population. Of these increases observed, changes in age distribution in both the entire and male cohorts modestly contributed (7.7% and 14.0%, respectively) to LA rate difference 2008 and 2017. Interestingly, the male cohort’s contribution was almost twice that of the change in age distribution in the entire cohort. In both the entire and male cohorts, changes in age-specific rates (92.3% and 86.0%, respectively) contributed to the largest share of the LA rate increase in the ten-year interval.

We also found that incorporating non-linear effects of covariates including age using GAM helped improve the model’s overall fit. The risk of LA significantly increased as age increases. In addition, the fixed effect of sex revealed that males were at a 2.4-fold greater risk of LA than females. In terms of the male-female case-to-population ratio, the male LA rate was 2.1–2.2 times higher than the female.

The increase in the province population by approximately 13.1 (rate of 1.31% annually) from 2008 to 2017 coincided with an increase in LA rate of 9.0 per 100,000 during the same time interval. The age group (65–74 years) had the largest percent increase in population (31.64%) and yielded the largest contribution of change in age distribution but the lowest contribution of change in age-specific rate. The age group 75+ years had the lowest percent increase in population (0.96%) in the decade and made the lowest contribution of changes in age but made a substantial contribution to the age-specific rate difference. These findings clearly show that when a population changes, the ripple effect of either the change in age distribution or age-specific rate impacts the population’s LA rate.

Changes in age structural transition and age-specific rate do not occur in isolation but come with changes in the rate of the age-related leading cause of LA, including vascular disease associated with diabetes [24, 25] and trauma (LA due to accident or injury) [7]. For a period of thirty-four years (1980–2014), global diabetes cases increased from 108 million to 422 million, of which 39.7% resulted from a rise in population, aging, and 31.8% from the interaction of the aging and rise in diabetes prevalence [25]. This evidence was similar to changes in the prevalence of diabetes in Saskatchewan over the years investigated. In both 2008 and 2017, the Canadian Community Health survey revealed that Saskatchewan’s diabetes prevalence was “about the same as Canada’s average” (5.9% and 7.3%, respectively) [26, 27]. Inferring from these estimates show that from 2008 to 2017, diabetes incidence in Saskatchewan increased by about 23.73%. This highlights the urgent need for strategized programs to respond to these changes in the foreseeable future. This is because Saskatchewan’s population is projected to grow 1.4 million, and the prevalence of diabetes to increase by 31% from 2020–2030 [28, 29].

Furthermore, two Canadian studies; one based on the present data and the other based on Canadian national data, found diabetes as the leading cause of LA in Canada and in Saskatchewan, with a prevalence of 65–80% [7, 30], respectively. Per our study data, the second leading cause of LA in Saskatchewan was PVD, followed by trauma [30]. This clearly indicates that besides diabetes, the impact of PVD and trauma on LA incidence in Saskatchewan cannot be underestimated [30]. In contrast, in other jurisdictions, trauma accounted for most of the LA incidence [31]. Sarvestani and Azam found trauma (54.2%) to be the leading cause of LA in Iran, followed by diabetes (26.4%) and vascular disease (10.5%) [31]. All the causes of LA highlighted above are age-dependent, diabetes and vascular disease are more common among individuals aged 60+years while trauma is more prevalent in individuals aged ≤39 years [32]. This demonstrates how the demographic shift in factors such as age could substantially impact the incidence of diabetes, vascular disease, trauma, and ultimately LA incidence.

Also, Siddiqui and Aziz’s study which focused on non-communicable diseases in Saskatchewan found that the trend of diabetes escalates till the age of 64 years [33], which is consistent with the higher contribution of age-specific LA rate experienced by individuals aged 50–64 in the entire population. In addition, in Saskatchewan, both the LA rate and incidence of diabetes are higher in First Nation persons than in the general population [34, 35]. However, the First Nations diabetes incidence rate declined at age ≥ 70 years [34]. Hence, the higher contribution of the age-specific LA rate in the entire population aged 50–64 years could in part be attributed to the increased diabetes incidence rate among First Nations persons aged 50–69 years [34].

More so, the decomposition procedure used for the present study [1] shows that the contribution of a particular age composition group for two given years (e.g., 2008 and 2017) partly relies on the multiplicative effect of the age-specific rate composition of that age group, whereas the contribution of a particular age-specific rate depends on the multiplicative effect of the age composition of that particular age group. The multiplicative effect of the increase in both age-specific LA rate and age composition absolute difference observed in persons aged 50–64 years in the entire cohort accounted for the higher age-specific LA rate witnessed in that group.

Males, on the other hand, witnessed a higher contribution of the shift in age composition than females and this is in part attributable to the higher age-specific rates and a marginal increase in age compositions in males than in females over the study period. Also, males, especially those aged ≤50 years in Canada, have a higher prevalence of diabetes than females [36], with the incidence of LA, reported to increase after age 55 years [37]. Siddiqui and Aziz’s study findings revealed that between 2003 and 2013, the diabetes prevalence rate in males in Saskatchewan rapidly increased from 5.4–9.2 compared to 5.7–6.1 per 100 population in females [33]. In addition, in both Saskatchewan and Canada, motor vehicle traffic accidents were more prevalent in males than in females [33].

The male cohort also saw a disproportionate contribution of both shifts in age distribution and age-specific LA rate. The absolute LA rate difference witnessed in persons aged 75+ years was the highest (210–127.32 = 82.68 per 100,000) compared to their counterparts in the other age groups. This contributed substantially to the higher age-specific LA rate contribution observed in persons aged 75+ years in the male cohort. Further, in Canada, males aged 75+ years have the highest prevalence of diabetes than their counterparts in the other age groups [38].

Although there was a statistically significant association between LA and all continuous covariate investigated via Poisson generalized additive model with linear effect of age and year of amputation (*Model II*), the present study has reiterated that using linear models on non-linear continuous covariate could undermine the performance of the fitted model [12]. A comparison of the Poisson GAM *Model I* and *Model II* in the present study based on the AIC and the analysis of deviance residuals revealed that incorporating the non-linear effect of age in LA-related study via GAM considerably improved the model fit than modeling age as a linear fixed effect. The latter finding was supported by Gavgani et al., who found that modeling non-linear effect of covariates using GAM significantly improved the model fit’s quality [12]. The risk of LA significantly increased as age increases in the present study is supported by previously published studies [39, 40].

Even though there was a marginal population difference between males and females in a decade in the present study, females had a reduced risk of LA than males. This was consistent with Cai et al. findings that an increased proportion of women veterans from 2008 to 2018 also led to a decrease incidence of lower extremity amputation [8]. Further, the present study’s higher case-to-population ratio of LA in males than females was supported by Kim et al.’s LA case ratio of 4.3 in men to 1 in women [32].

### Strengths and limitations

To the best of our knowledge, this is the first time the innovative method of decomposition analysis and robust GAM models have been used to assess the contribution of population and age structural transition and the non-linear effect of age on LA incidence in a population-based study. The study also had a limitation. Thus, only limited covariates were available to be included in the study models.

## Conclusion

Understanding the current impact of changing demographics on disease incidence provides the best opportunity to plan into the future. In a decade, we found that changes in age distribution and age-specific rate substantially impacted the increase in the LA rate observed in the province. This highlights the urgent need for strategized programs to respond to these changes as both the population and diabetes, which is age-dependent and a leading cause of LA, are expected to increase in the province by 2030. As changes in population and demographic factors are inevitable, this study provides data for policy makers on the need for continuous incorporation of the shift in population in the design of future health services. In addition, we found that in amputation-related research, incorporating non-linear effects of age via GAM could considerably improve the model fit’s quality.
